# Exhibition by International Tennis Players

**Published:** 1924-08

**Authors:** 


### Exhibition by International Tennis Players.

Among the many activities on the part of King's College Hospital for making money for the hospital there was held, on July 7th, in the hospital grounds, an exhibition of tennis by the international players, Miss McKane, Mrs. Covell (better known as Miss Howkins), Mr. J. B. Gilbert, and Mr. L. A. Godfree. Every available seat was filled from the chairs around the courts to the flat roofs and balconies from which the students watched. Although we must not look at a gift-game too critically and fully appreciate the kindness of the players in giving the exhibition so soon after their hard week of championship play, the games struck us as being more leisurely than we should expect, and wish, to see by such famous players. In the first match, J. B. Gilbert—who, by the way, is a left-handed player—beat L. A. Godfree 6—4, 6—1. In the set between Miss McKane and Mrs Covell, Mrs. Covell won, in a very close match, 6—5

